# Gut dysbiosis impairs intestinal renewal and lipid absorption in Scarb2 deficiency-associated neurodegeneration

**DOI:** 10.1093/procel/pwae016

**Published:** 2024-04-18

**Authors:** Yinghui Li, Xingchen Liu, Xue Sun, Hui Li, Shige Wang, Wotu Tian, Chen Xiang, Xuyuan Zhang, Jiajia Zheng, Haifang Wang, Liguo Zhang, Li Cao, Catherine C L Wong, Zhihua Liu

**Affiliations:** Institute for Immunology and School of Basic Medicine, Tsinghua University, Beijing 100084, China; Key Laboratory of Infection and Immunity, Institute of Biophysics, Chinese Academy of Sciences, Beijing 100101, China; Institute of Biophysics, University of Chinese Academy of Sciences, Beijing 100049, China; Institute for Immunology and School of Basic Medicine, Tsinghua University, Beijing 100084, China; Tsinghua-Peking Center for Life Sciences, Tsinghua University, Beijing 100084, China; First School of Clinical Medicine, Peking University First Hospital, Peking University, Beijing 100034, China; School of Pharmacy, University of Wisconsin-Madison, Madison, WI 53705, United States; Key Laboratory of Infection and Immunity, Institute of Biophysics, Chinese Academy of Sciences, Beijing 100101, China; Institute of Biophysics, University of Chinese Academy of Sciences, Beijing 100049, China; Department of Neurology, Shanghai Jiao Tong University Affiliated Sixth People’s Hospital, Shanghai 200233, China; Department of Neurology, Shanghai Jiao Tong University Affiliated Sixth People’s Hospital, Shanghai 200233, China; Key Laboratory of Infection and Immunity, Institute of Biophysics, Chinese Academy of Sciences, Beijing 100101, China; Institute of Biophysics, University of Chinese Academy of Sciences, Beijing 100049, China; Key Laboratory of Infection and Immunity, Institute of Biophysics, Chinese Academy of Sciences, Beijing 100101, China; Department of Laboratory Medicine, Peking University Third Hospital, Beijing, 100191, China; Clinical Stem Cell Research Center, Peking University Third Hospital, Beijing 100191, China; Key Laboratory of Infection and Immunity, Institute of Biophysics, Chinese Academy of Sciences, Beijing 100101, China; Department of Neurology, Shanghai Jiao Tong University Affiliated Sixth People’s Hospital, Shanghai 200233, China; Department of Medical Research Center, State Key Laboratory of Complex Severe and Rare Diseases, Peking Union Medical College Hospital, Chinese Academy of Medical Sciences & Peking Union Medical College, Beijing 100730, China; Tsinghua University-Peking University Joint Center for Life Sciences, Peking University, Beijing 100084, China; Institute for Immunology and School of Basic Medicine, Tsinghua University, Beijing 100084, China; Tsinghua-Peking Center for Life Sciences, Tsinghua University, Beijing 100084, China

**Keywords:** gut dysbiosis, intestinal renewal, neuron disease

## Abstract

Scavenger receptor class B, member 2 (SCARB2) is linked to Gaucher disease and Parkinson’s disease. Deficiency in the SCARB2 gene causes progressive myoclonus epilepsy (PME), a rare group of inherited neurodegenerative diseases characterized by myoclonus. We found that Scarb2 deficiency in mice leads to age-dependent dietary lipid malabsorption, accompanied with vitamin E deficiency. Our investigation revealed that Scarb2 deficiency is associated with gut dysbiosis and an altered bile acid pool, leading to hyperactivation of FXR in intestine. Hyperactivation of FXR impairs epithelium renewal and lipid absorption. Patients with SCARB2 mutations have a severe reduction in their vitamin E levels and cannot absorb dietary vitamin E. Finally, inhibiting FXR or supplementing vitamin E ameliorates the neuromotor impairment and neuropathy in Scarb2 knockout mice. These data indicate that gastrointestinal dysfunction is associated with SCARB2 deficiency-related neurodegeneration, and SCARB2-associated neurodegeneration can be improved by addressing the nutrition deficits and gastrointestinal issues.

## Introduction

Scarb2 is a member of the scavenger receptor family, which includes CD36, Scarb1, and Scarb2 (Calvo et al., 1995). Scarb2 acts as the mannose-6-phosphate-independent trafficking receptor for β-glucocerebrosidase (β-GCase), a lysosomal enzyme that is deficient in most cases of Gaucher disease (GD) (Reczek et al., 2007). Loss of functional Scarb2 leads to mistargeting of β-GCase to the extracellular space, rather than lysosomes (Reczek et al., 2007). Loss of Scarb2 leads to alpha-synuclein dependent neurodegeneration in mice (Rothaug et al., 2014), consistent with the role of β-GCase in the development of synucleinopathies (Mazzulli et al., 2011). Genetically, deficiency of SCARB2 is a risk factor in GD and Parkinson’s disease (PD) (Blauwendraat et al., 2019; Michelakakis et al., 2012; Velayati et al., 2011), while complete loss of function of SCARB2 underlies severe progressive myoclonic epilepsy (PME-4) (Tian et al., 2018).

Abnormal body weight and related metabolic disorders are common in both GD and PD patients (Barichella et al., 2009; Kałużna et al., 2019; Wills et al., 2017). In facts, weight loss observed in PD patients has been linked to accelerated motor and cognitive decline (Urso et al., 2022). Changes in the nervous system, such as loss of appetite and involuntary movement, has been believed to contribute to the weight loss, however, other factors influencing metabolism, such as neurodegeneration-associated gut dysbiosis, have not been thoroughly investigated.

Gut dysbiosis occurs in neurodegenerative conditions, such as Alzheimer’s disease, PD and amyotrophic lateral sclerosis (Marizzoni et al., 2017). Increasing amounts of evidence suggest a key role of the gut–brain axis in development of neurodegenerative disorders. PD is noted for a high prevalence of gastrointestinal dysfunction (Warnecke et al., 2022). There is no report on gut microbiota in patients with GD or PME-4. Although the root cause of the dysbiosis remains unknown, it is believed that the gut dysbiosis may impair the barrier function of the intestine and augment inflammation. However, the molecular mechanisms by which dysbiosis leads to impaired intestinal function remain to be determined.

The bile acid (BA)-microbiome feedback loop is increasingly recognized for its role in mediating host-microbe crosstalk (Collins et al., 2023; Zhao et al., 2023). The BA pool is composed of both primary and secondary BAs. Primary BAs are synthesized by hepatocytes from cholesterol and can be further modified with glycine or taurine. In humans, the primary BAs are cholic acid (CA) and chenodeoxycholic acid (CDCA), and in mice, CA and muricholic acid (MCA). Gut bacteria metabolize primary bile acids in the colon to produce secondary BAs. This transformation process consists of four distinct steps: deconjugation of the amino acid glycine or taurine, and dehydroxylation, dehydrogenation, and epimerization of the cholesterol core. In this way, intestinal microbes greatly enhance the BA diversity.

The Farnesoid X Receptor (FXR) is a major receptor for BAs. Most endogenous BAs act as agonists while certain conjugated BAs, such as tauro-alpha (T-α) or tauro-beta (T-β) muricholic acid (MCA), are strong antagonists (Sayin et al., 2013). FXR is widely expressed in many tissues. FXR signaling is best understood in enterocytes and hepatocytes. FXR signaling plays an important role in liver–intestine crosstalk. FXR in hepatocytes regulates BA synthesis by inducing expression of the transcriptional repressors SHP (small heterodimer partner) and MafG (V-Maf avian musculoaponeurotic fibrosarcoma oncogene homolog G), which in turn represses the rate-limiting genes involved in BA synthesis (de Aguiar Vallim et al., 2015; Kerr et al., 2002; Wang et al., 2002). Activation of FXR in enterocytes induces FGF15 in mouse, and FGF19 in human, which act in an endocrine manner to activate FGFR4 in hepatocytes, limiting BA synthesis (Inagaki et al., 2005). In addition to crosstalk with the liver, FXR in enterocytes also regulate various aspects of intestinal physiology. For instance, activation of FXR in the intestine limits lipid absorption during the development of non-alcoholic fatty liver disease (Clifford et al., 2021). Intestinal FXR inhibition decreases ceramide levels by suppressing ceramide synthesis specifically in the enterocytes (Jiang et al., 2015a). Antagonism of FXR by BAs induces proliferation and DNA damage in Lgr5^+^ cells, influencing the progression of colorectal cancer (Fu et al., 2019). Despite all these progresses, the role of FXR in dysbiosis associated with neurodegeneration remains largely uninvestigated.

Here, we found the Scarb2-associated neurodegeneration leads to a progressive defect in dietary fat absorption. Mechanistically, profound gut dysbiosis in Scarb2 knockout mice results in altered BA-FXR signaling and impaired intestinal function. Finally, addressing the malnutrition mitigates the neurodegeneration in Scarb2 knockout mice.

## Results

### Progressive loss of body fat in *Scarb2*^−/−^ mice

**Figure 1.**
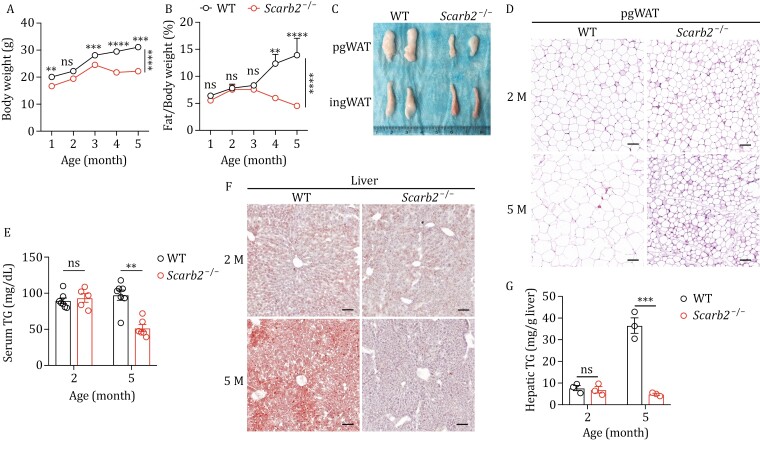
Progressive lipodystrophy in ***Scarb2*^–/–^** mice. (A) Body weight of WT and *Scarb2*^–/–^ mice at different ages (*n* = 5–6 mice per group). (B) The ratio of body fat of WT and *Scarb2*^–/–^ mice at different ages (*n* = 5–7 mice per group). (C) Representative images of isolated perigonadal white adipose tissue (pgWAT) and inguinal white adipose tissue (ingWAT) from 5-month-old WT and *Scarb2*^–/–^ mice. (D) Representative images of H&E-stained sections of pgWAT from 2-month-old (2M) and 5-month-old (5M) WT and *Scarb2*^–/–^ mice. (E) The level of serum TG of 2-month-old and 5-month-old WT and *Scarb2*^–/–^ mice (*n* = 5–7 mice per group). (F) Representative images of Oil Red O staining of liver from 2-month-old and 5-month-old WT and *Scarb2*^–/–^ mice. (G) Abundance of hepatic TG in 2-month-old and 5-month-old WT and *Scarb2*^–/–^ mice. Scale bars, 100 μm in (D and F). Mean values are plotted with bars indicating SEM in (A and B). Means (±SEM) are plotted with each symbol representing an individual animal in (E and G). *P* values were calculated with two-way ANOVA with the Bonferroni correction for multiple tests in (A and B) or multiple *t* tests in (E and G). “ns” indicates no significant difference (*P *> 0.05). **P < 0.01, ***P < 0.001, ****P < 0.0001.

Consistent with a previous report that *Scarb2* knockout led to severe neuromotor impairments, such as tremor and hindlimb clasping (Rothaug et al., 2014), we observed similar neuromotor impairment phenotypes in *Scarb2*^−/−^ mice from 3 months of age (Fig. S1). Accompanying the progression of neuromotor impairment, *Scarb2*^−/−^ mice failed to gain more weight from the age of 3 months (Fig. 1A). This observation is consistent with the previous publications (Rothaug et al., 2014; Zou et al., 2022). EchoMRI indicated a significantly lower fat mass in *Scarb2*^−/−^ mice from 3 months of age (Fig. 1B). The gonadal fat and inguinal fat tissues were smaller in 5-month-old *Scarb2*^−/−^ mice than in WT mice (Fig. 1C). The size of individual adipocytes was reduced in *Scarb2*^−/−^ mice, especially in 5-month-old mice (Fig. 1D). The level of serum triglyceride (TG) was significantly lower in 5-month-old *Scarb2*^−/−^ mice than in WT mice (Fig. 1E). Oil-red-O analysis of liver tissue showed less accumulation of oil droplets in 5-month-old *Scarb2*^−/−^ mice compared to WT control (Fig. 1F). Accordingly, the liver TG content in 5-month-old *Scarb2*^−/−^ mice was significantly lower than that in WT (Fig. 1G). There were no significant differences in TG level in serum or liver when mice were at the age of 2 months (Fig. 1E and 1G). Thus, *Scarb2*^−/−^ mice started progressive loss of body fat from 3 months of age.

### Impaired dietary fat absorption in old *Scarb2*^−/−^ mice

Considering the progressive loss of body fat in *Scarb2*^−/−^ mice, we asked whether neuromotor impairment might affect food intake and metabolic rate. *Scarb2*^−/−^ mice had comparable food intake with WT at different ages (Fig. S2). At 2 months of age, *Scarb2*^−/−^ mice had comparable energy expenditure with WT mice; however, at 5 months of age, *Scarb2*^−/−^ mice had significantly higher energy expenditure than WT mice (Fig. S2). There was no different in respiratory exchange ratio (RER) between WT and *Scarb2*^−/−^ mice, which indicates no difference in preferred energy source, such as fat or carbohydrate.

**Figure 2.**
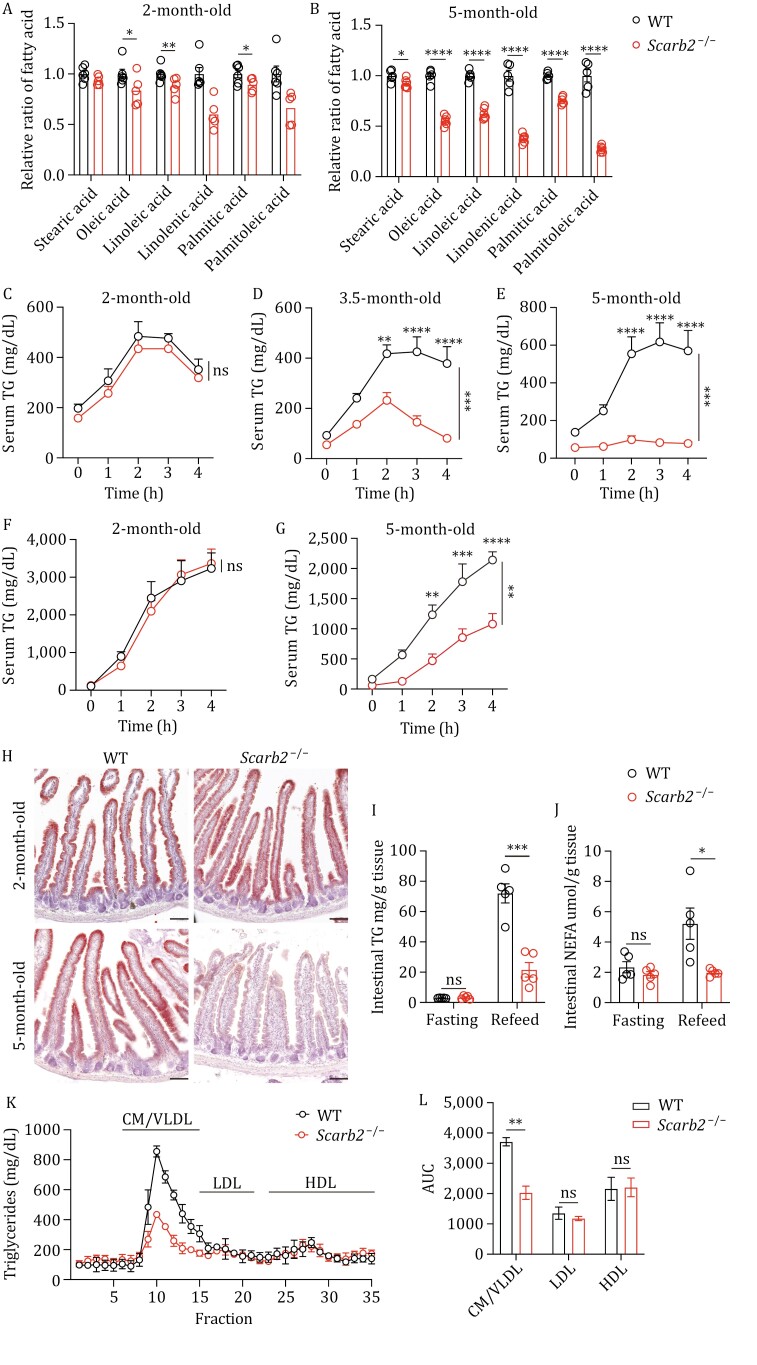
Impaired dietary fat absorption in old ***Scarb2*^–/–^** mice. (A and B) Relative levels of different fatty acids present in saponified plasma samples from WT and *Scarb2*^–/–^ mice at 2 months (A) or 5 months (B). The levels of different fatty acids were all normalized to the level of WT mice (*n* = 5–6 mice per group). (C–E) Serum TG concentration of 2-month-old (C), 3.5-month-old (D) and 5-month-old (E) WT and *Scarb2*^–/–^ mice after an acute dietary fat challenge (*n* = 4–8 mice per group). (F and G) Serum TG concentration of 2-month-old (F) and 5-month-old (G) WT and *Scarb2*^–/–^ mice which were injected with the lipoprotein lipase inhibitor tyloxapol, then subjected to an acute dietary fat challenge (*n* = 5–6 mice per group). (H) Representative images of Oil Red O staining of duodenum from 2-month-old and 5-month-old WT and *Scarb2*^–/–^ mice after olive oil gavage. (I and J) Effect of 12-h food deprivation (Fasting) and 2-h refeeding through oral gavage of olive oil (refeed) on the levels of triglycerides (I) and NEFA (J) in duodenum of 5-month-old WT and *Scarb2*^–/–^ mice (*n* = 5 mice per group). (K and L) Fractionation analysis of plasma lipids from mice 2 h after oil gavage using fast performance liquid chromatography. TG concentrations in each fraction (K) and the calculated area under the curve (AUC) for indicated fractions (L) (*n* = 3 mice per group). Scale bars, 100 μm in (H). Mean values with bars indicating SEM are plotted in (C–G, K and L). Means (±SEM) are plotted with each symbol representing an individual animal in (A, B, I, and J). *P* values were calculated using two-way ANOVA with the Bonferroni correction for multiple tests in (C–G) and multiple *t* tests in (A, B, I, J and L). CM, chylomicrons; VLDL, very-low-density lipoproteins; LDL, low-density lipoproteins; HDL, high-density lipoproteins. “ns” indicates no significant difference (*P *> 0.05). *P < 0.05, **P < 0.01, ***P < 0.001, ****P < 0.0001.

The involvement of the scavenger receptor protein family in lipid metabolism prompted us to investigated whether Scarb2 deficiency might affect dietary fat absorption. Analysis of the fatty acid composition in alkali-saponified plasma indicated a decreased abundance of essential fatty acids in 5-month-old *Scarb2*^−/−^ mice relative to WT mice (Fig. 2A and 2B). We next evaluated the absorption of dietary fat by monitoring the postprandial triglyceridemic response in WT and *Scarb2*^−/−^ mice. At 2 months of age, *Scarb2*^−/−^ mice had similar dynamics of serum TG concentration as WT (Fig. 2C). However, the postprandial peak of serum TG in *Scarb2*^−/−^ mice was significantly lower than that in WT mice at the age of 3.5 months (Fig. 2D). The difference became more pronounced when the animals were 5 months old (Fig. 2E). To evaluate the net effect of dietary fat absorption, serum TG concentration was monitored after an acute dietary fat challenge in the presence of tyloxapol to inhibit lipase activity in the circulation. Two-month-old WT and *Scarb2*^−/−^ mice had comparable levels of serum TG at different time points after the acute dietary fat challenge (Fig. 2F). The level of serum TG in 5-month-old *Scarb2*^−/−^ mice was significantly lower than that in WT mice (Fig. 2G). Oil-red-O staining was comparable between 2-month-old *Scarb2*^−/−^ and WT mice, but was markedly diminished in 5-month-old *Scarb2*^−/−^ mice compared to WT mice (Fig. 2H). The levels of TG and nonesterified fatty acid (NEFA) were quantified, and 5-month-old *Scarb2*^−/−^ mice had significantly lower levels of TG and NEFA in intestine after oil feeding (Fig. 2I and 2J). Accordingly, plasma lipoprotein profiles revealed significantly reduced TG in chylomicron (CM)/very-low-density lipoprotein (VLDL) fractions, but not in low-density lipoprotein (LDL) or high-density lipoprotein (HDL) fractions, in 5-month-old *Scarb2*^−/−^ mice 2 h after oil gavage (Fig. 2K and 2L). Collectively, these data indicate a profound defect in absorption of dietary fat in aged but not in younger *Scarb2*^−/−^ mice, which argues against a direct role of Scarb2 in fat absorption.

### Profound gut dysbiosis and altered bile acids in old *Scarb2*^−/−^ mice

**Figure 3.**
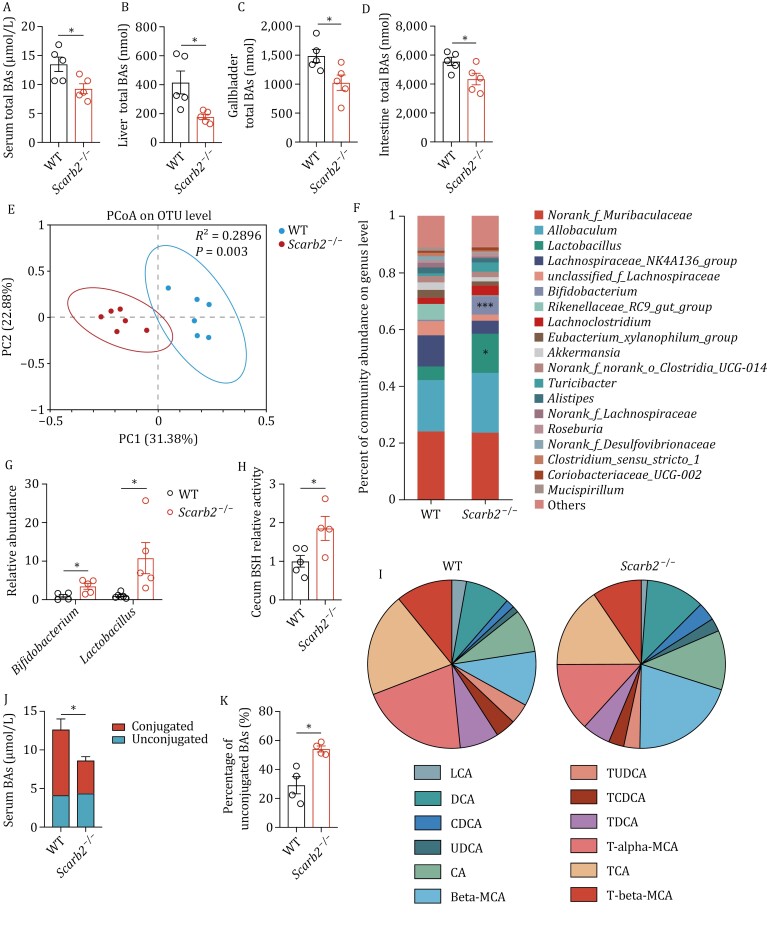
Gut dysbiosis and alterations of bile acids (BAs). (A–D) BA concentration in serum (A) and total bile acid content of liver (B), gallbladder (C), and intestine (D) in 5-month-old WT and *Scarb2*^–/–^ mice (*n* = 5 mice per group). (E) PCoA analysis of fecal microbiota based on the relative abundance of bacterial OTUs (*n* = 6 mice per group). (F) Relative genus abundance in the feces of separately housed 5-month-old WT and *Scarb2*^–/–^ mice (*n* = 6 mice per group). (G) Relative abundance of bacteria in the genera *Bifidobacterium* and *Lactobacillus* in fecal microbiota of 5-month-old WT and *Scarb2*^–/–^ mice (*n* = 5 mice per group). (H) Cecum BSH activity in 5-month-old WT and *Scarb2*^–/–^ mice (*n* = 4–5 mice per group). (I) Serum bile acid composition in 5-month-old WT and *Scarb2*^–/–^ mice (*n* = 4 mice per group). (J and K) The concentration of conjugated and unconjugated BAs (J) and the percentage of unconjugated BAs (K) in 5-month-old WT and *Scarb2*^–/–^ mouse sera (*n* = 4 mice per group). Means (±SEM) are plotted with each symbol representing an individual animal in (A–D, G, H and K). Means with bars indicating SEM are plotted in (J). *P* values were calculated using two-tailed Student’s *t*-tests in (A–D, H, J, and K) and multiple *t* tests in (G). “ns” indicates no significant difference (*P *> 0.05). *P < 0.05, ***P < 0.001.

The digestion and absorption of dietary fat relies on the collective action of pancreatic lipases and bile acids. 5-month-old *Scarb2*^−/−^ mice had a comparable level of lipase activity with control mice (Fig. S3A and S3B). Gallbladders in 5-month-old *Scarb2*^−/−^ mice were shrunken in size compared to WT (Fig. S3C). Accordingly, there were reduced levels of bile acids in liver, serum, gallbladder, and intestine in 5-month-old *Scarb2*^−/−^ mice (Fig. 3A–D).

Bile acids play an important role in modulating the microbiota. Total 16s rDNA quantification showed a higher bacterial load in 5-month-old *Scarb2*^−/−^ mice compared to WT mice (Fig. S3D). 16s rDNA sequencing was performed on fecal samples from 5-month-old animals. Fecal microbiota showed similar alpha-diversity in WT and *Scarb2*^−/−^ mice (Fig. S3E and S3F). Principal coordinate analysis (PCoA) at the operational taxonomic unit (OTU) level showed significant differences between WT and *Scarb2*^−/−^ mice (Fig. 3E). Bacteria in the genera *Lactobacillus* and *Bifidobacterium* were significantly more frequent in 5-month-old *Scarb2*^−/−^ mice (Fig. 3F and 3G). *Lactobacilli* and *Bifidobacteria* deconjugate bile acids in the gastrointestinal tract through the action of the enzyme bile salt hydrolase (BSH) (Tanaka et al., 1999). Indeed, BSH activity was enhanced in cecum in *Scarb2*^−/−^ mice (Fig. 3H). Profiling of bile acids showed differences in BA composition (Fig. 3I). The abundance of conjugated BAs was reduced in serum (Fig. 3J), with unconjugated bile acids being the dominant type in 5-month-old *Scarb2*^−/−^ mice (Fig. 3K).

To ascertain whether dysbiosis associated with Scarb2 deficiency is sufficient to induce fat malabsorption, we transferred cecum microbiota from 5-month-old WT or *Scarb2*^–/–^ mice to WT recipients. Subsequently, we assessed the ability of these mice to absorb dietary fats. Mice that received microbiota from *Scarb2*^–/–^ mice demonstrated lower serum triglyceride concentrations compared to those that received microbiota from WT mice (Fig. S3G).

### Altered BA-FXR signaling decreases BA synthesis and impairs epithelium renewal

**Figure 4.**
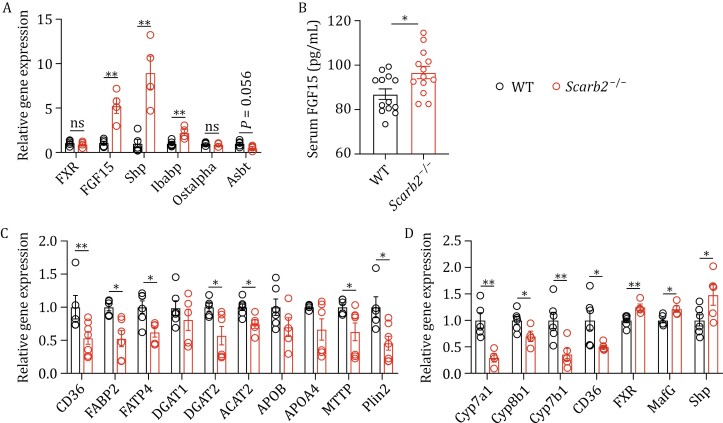
Elevated FXR-related gene expression and decreased lipid absorption-related gene expression in ***Scarb2*^–/–^** mice. (A) Relative mRNA levels of FXR-related genes in jejunum of 5-month-old WT and *Scarb2*^–/–^ mice (*n* = 4–5 mice per group). (B) Serum FGF15 concentration of 5-month-old WT and *Scarb2*^–/–^ mice (*n* = 13 mice per group) (C and D) Relative mRNA levels of genes related to jejunum (C) and liver (D) lipid metabolism and bile acids production in 5-month-old WT and *Scarb2*^–/–^ mice 2 h after oral gavage of olive oil (*n* = 5 mice per group). Means (±SEM) are plotted with each symbol representing an individual animal in (A–D). *P* values were calculated using multiple *t* tests in (A, C, and D). *P* values were calculated using two-tailed Student’s *t*-tests in (B). “ns” indicates no significant difference (*P* > 0.05). *P < 0.05, **P < 0.01.

FXR is a transcription factor that executes BA-induced transcriptional programs. Intestinal FXR activation induces FGF15 in the intestine to potently inhibit *de novo* BA synthesis in the liver (Kliewer et al., 2015). We suspected that the altered pool of bile acids in *Scarb2*^−/−^ might alter the BA-FXR-FGF15 signaling. The Fgf15 mRNA level was significantly elevated in intestine in 5-month-old *Scarb2*^−/−^ mice, indicating higher activity of FXR (Fig. 4A). Serum FGF15 protein level also increased in *Scarb2*^−/−^ mice (Fig. 4B). Accordingly, small heterodimer partner (Shp), a major target gene of FXR, was upregulated at the mRNA level in small intestine (Fig. 4A). Intestinal Shp has been reported to suppress the expression of genes involved in fatty acid uptake (Seok et al., 2022). Thus, we determined the expression levels of key genes involved in the fatty acid uptake process, such as CD36, FABPs, and DGATs. Our results showed that mRNA levels of these genes were all downregulated in jejunum in 5-month-old *Scarb2*^−/−^ mice (Fig. 4C).

FGF15 produced in the small intestine signals through FGFR4 in hepatocytes to inhibit expression of the liver CYP7A1 gene (Kliewer et al., 2015). The CYP7A1 enzyme catalyzes the rate-limiting step in the synthesis of BAs from cholesterol. We found that Cyp7a1 mRNA was downregulated in liver in 5-month-old *Scarb2*^−/−^ mice (Fig. 4D). Cyp8b1 and Cyp7b1 mRNA levels were also reduced in liver in 5-month-old *Scarb2*^−/−^ mice (Fig. 4D), which likely reflects the overall inhibition of classic BA synthesis. MafG and Shp, the suppressors of BA synthesis, were both upregulated at the mRNA level in livers in 5-month-old *Scarb2*^−/−^ mice (Fig. 4D).

**Figure 5.**
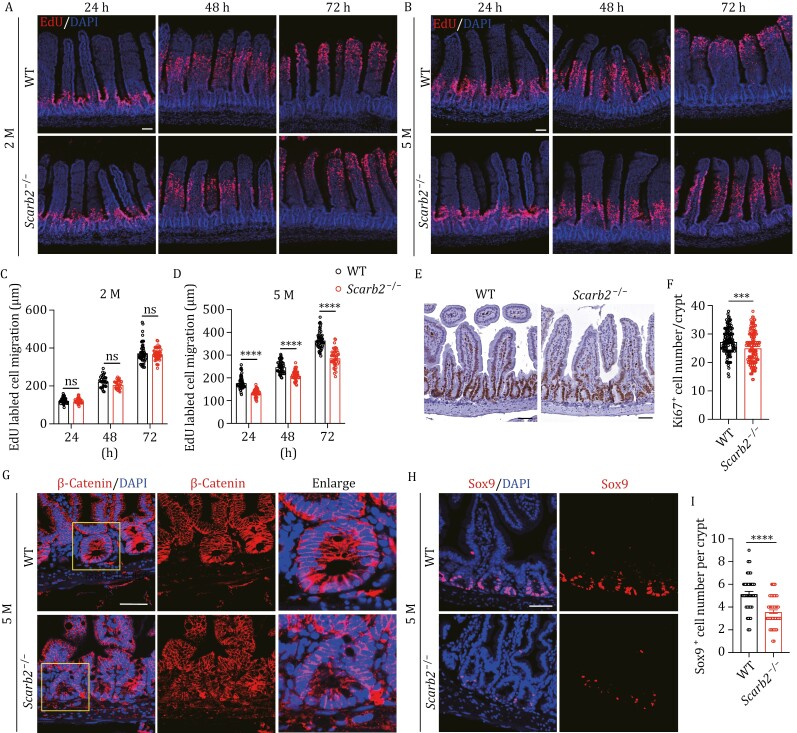
Scarb2 deficiency impairs intestinal cell proliferation. (A and B) Representative confocal images of cell migration in 2-month-old (A) and 5-month-old (B) WT and *Scarb2*^–/–^ mice jejunum at indicated time points after EdU injection. Images are representative of three independent experiments (*n* = 3 mice per group). (C and D) Distance migrated by EdU-labeled cells in 2-month-old (C) and 5-month-old (D) WT and *Scarb2*^–/–^ mice jejunum. The migration distance was measured from the bottom of the crypt to the furthest EdU-labeled cells (*n* = 3 mice per group, 5–6 fields per mouse, 2–3 villus per fields). (E) Representative images of Ki67 staining in intestine of 5-month-old WT and *Scarb2*^–/–^ mice. (F) Quantification of Ki67^+^ cell number in intestine of 5-month-old WT and *Scarb2*^–/–^ mice (*n* = 5 mice per group, 5–10 fields per mouse, 2–3 crypt per fields). (G and H) Representative confocal images of β-catenin (G) and Sox9 (H) staining in jejunum of 5M-old WT and *Scarb2*^–/–^ mice. (I) Quantification of Sox9^+^ cell number per crypt in jejunum of 5-month-old WT and *Scarb2*^–/–^ mice (*n* = 5 mice per group, 3–4 fields per mouse, 2–3 crypt per fields). Nuclei were counterstained with DAPI (A, B, G, and H). Scale bars, 50 μm in (A, B, E, G, and H). Means (±SEM) are plotted with each symbol representing an individual data point in (C, D, F, and I). *P* values were calculated using two-tailed Student’s *t*-tests in (F and I) and two-way ANOVA with the Bonferroni correction for multiple tests in (C and D). “ns” indicates no significant difference (*P *> 0.05). *P < 0.05, **P < 0.01, ***P < 0.001, ****P < 0.0001.

In intestine, FXR intertwines with Wnt signaling, which has been implicated in colon tumorigenesis (Fu et al., 2019). We were prompted to determine whether Wnt signaling and intestinal renewal are impaired in *Scarb2*^−/−^ mice. Villus length and crypt depth in WT and *Scarb2*^−/−^ mice were comparable in 2-month-old animals, but were significantly decreased in 5-month-old *Scarb2*^−/−^ mice (Fig. S4A–D). The density of crypts was also reduced in 5-month-old *Scarb2*^−/−^ mice (Fig. S4E). These data indicated reduced epithelium renewal in *Scarb2*^−/−^ mice. Thus, we performed EdU pulse-chase experiments to evaluate the rate of epithelium turnover. EdU-labeled cells migrated significantly more slowly in 5-month-old *Scarb2*^−/−^ mice compared to age-matched controls (Fig. 5A–D). Ki67 staining also revealed fewer highly proliferating cells in 5-month-old *Scarb2*^−/−^ mice (Fig. 5E and 5F). To determine whether Wnt signaling was affected, we detected beta-catenin by immunostaining. Nuclear staining of beta-catenin was significantly reduced in *Scarb2*^−/−^ mice (Fig. 5G). Next, we immunostained Sox9, an intestine crypt transcription factor regulated by Wnt signaling. A significantly reduced number of Sox9^+^ cells was present in *Scarb2*^−/−^ mice (Fig. 5H and 5I).

**Figure 6.**
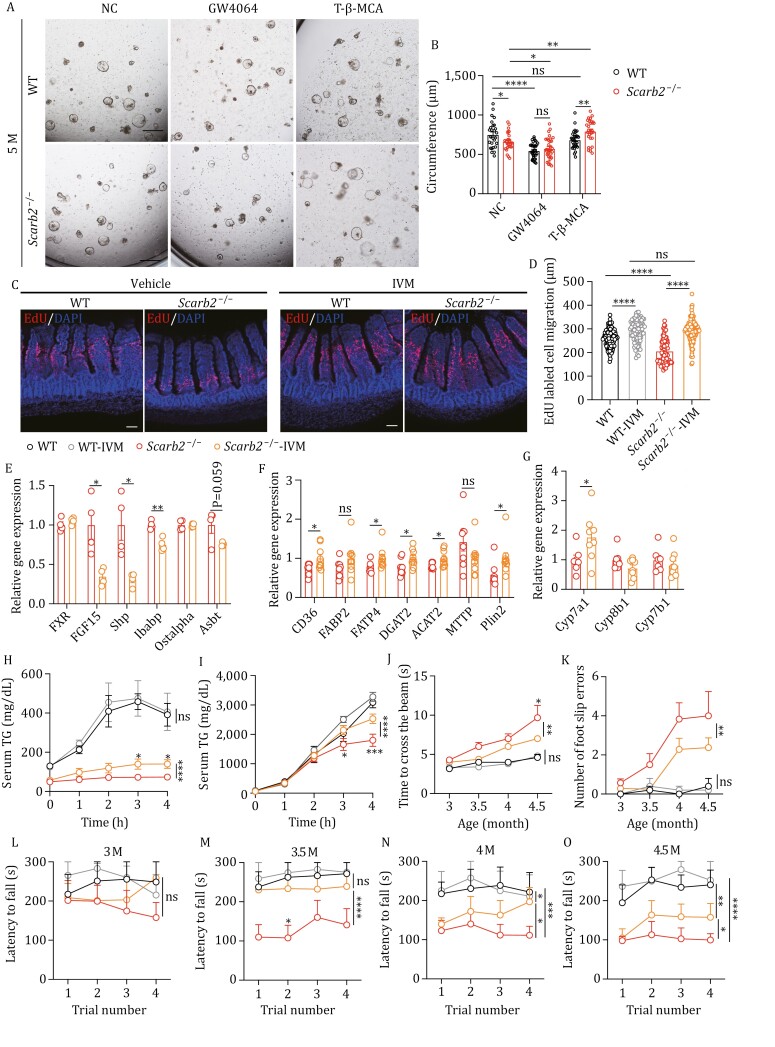
Inhibiting FXR rescues epithelium renewal and motor function in ***Scarb2*^–/–^** mice. (A and B) Representative images (A) and circumference quantification (B) of intestinal organoids by co-culturing isolated intestinal crypts from 5-month-old WT and *Scarb2*^–/–^ mice together with WT stromal cells and treated with GW4064 or T-β-MCA. (C) Representative confocal images of cell migration in jejunum in 5-month-old mice at 48 h after EdU injection. Images are representative of four independent experiments (*n* = 3 mice per group). (D) Migration distance of EdU-labeled cells in jejunum of 5-month-old WT and *Scarb2*^–/–^ mice treated with vehicle or IVM (*n* = 3 mice per group, 5–6 fields per mouse, 2–3 villus per fields). (E) Relative mRNA levels of FXR-related genes in jejunum of 5-month-old WT and *Scarb2*^–/–^ mice treated with vehicle or IVM (*n* = 4 mice per group). (F) Relative mRNA levels of genes related to lipid metabolism in jejunum of 5-month-old *Scarb2*^–/–^ mice treated with vehicle or IVM 2 h after oral gavage of olive oil (*n* = 8 mice per group). (G) Relative mRNA levels of FXR-related genes in liver of 5-month-old *Scarb2*^–/–^ mice treated with vehicle or IVM (*n* = 6–8 mice per group). (H) Serum TG concentration of 5-month-old WT and *Scarb2*^–/–^ mice treated with vehicle or IVM after an acute dietary fat challenge (*n* = 5–9 mice per group). (I) Serum TG concentration of 5-month-old WT and *Scarb2*^–/–^ mice treated with vehicle or IVM which were pretreated with the lipoprotein lipase inhibitor tyloxapol, subjected to an acute dietary fat challenge (*n* = 5–9 mice per group). (J and K) Time needed to cross the beam and number of foot slip errors by beam walk test in the indicated mice at different ages (*n* = 8 mice per group). (L–O) Latency to fall measured by rotarod test in the indicated mice at different ages (*n* = 8–9 mice per group). Scale bars, 500 μm in (A), 50 μm in (C). NC, negative control. Means (±SEM) are plotted with each symbol representing an individual data point in (B and D). Means (±SEM) are plotted with each symbol representing an individual animal in (E–G). Mean values with bars indicating SEM are plotted in (H–O). *P* values were calculated using multiple *t* tests in (E–G). *P* values were calculated using two-way ANOVA with the Bonferroni correction for multiple tests in (B, D, and H–O). “ns” indicates no significant difference (*P *> 0.05). *P < 0.05, **P < 0.01, ***P < 0.001, ****P < 0.0001.

The epithelium renewal is driven by intestinal stem cells localized in the crypts. We employed intestinal organoid culture to evaluate the renewal potential. We co-cultured freshly isolated crypts together with WT stromal cells to mimic the stem cell niche found in the intestine. The growth of such intestinal organoids, evaluated by the circumference, was significantly slower in organoids derived of crypts from 5-month-old *Scarb2*^−/−^ mice (Fig. 6A and 6B). In comparison, there was no difference in the growth of intestinal organoids derived from WT and *Scarb2*^−/−^ mice at 2 months (Fig. S4F and S4G). To determine whether FXR activation underlies the impaired renewal in 5-month-old *Scarb2*^−/−^ mice, we treated intestinal organoids with the FXR agonist GW4064 and the FXR antagonist tauro-beta-MCA (T-β-MCA). As expected, GW4064 treatment markedly reduced the growth of WT intestinal organoids (Fig. 6A and 6B). Interestingly, activation of FXR by GW4064 eliminated the difference between growth of WT and *Scarb2*^−/−^ organoids (Fig. 6A and 6B). FXR inhibition by T-β-MCA enhanced the growth of organoids derived from *Scarb2*^−/−^ mice, but had no effect on organoids derived from WT mice (Fig. 6A and 6B). This indicates that the growth defect of *Scarb2*^−/−^ organoids is due to FXR hyperactivation. We wanted to determine whether inhibiting FXR can rescue the epithelium renewal defect in *Scarb2*^−/−^ mice *in vivo*. Since T-β-MCA might be metabolized differently in WT and *Scarb2*^−/−^ mice, we used ivermectin (IVM), a highly selective FXR inhibitor (Jin et al., 2013), to evaluate the effect of inhibiting FXR on epithelium renewal. Remarkably, a course of 2-week IVM treatment effectively restored normal epithelial renewal in 5-month-old *Scarb2*^−/−^ mice (Fig. 6C and 6D).

**Figure 7.**
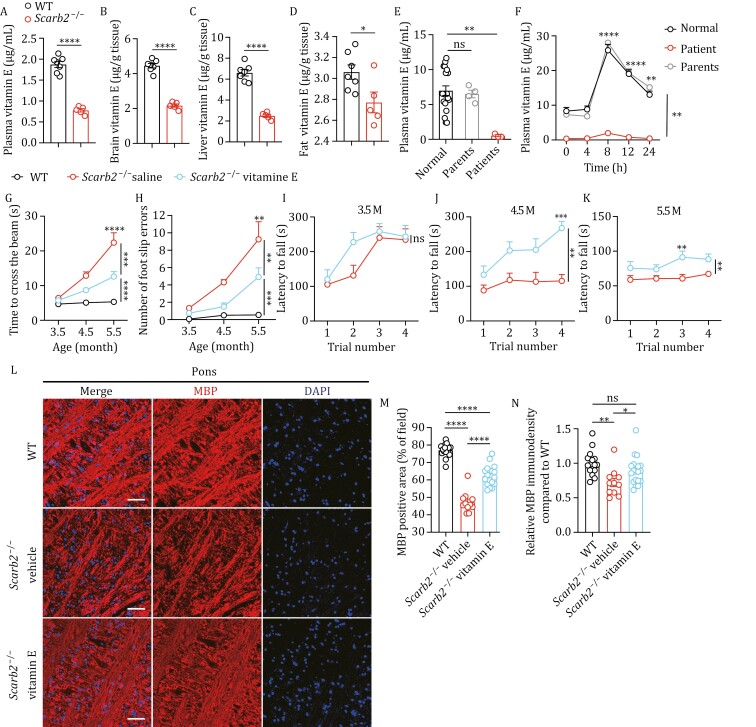
Deficiency of vitamin E in ***Scarb2*^–/–^** mice and patients with ***SCARB2*** mutation and supplementation of vitamin E ameliorates the neuromotor deficits. (A–D) Vitamin E concentration in plasma (A), brain (B), liver (C), and fat (D) from 5-month-old *Scarb2*^–/–^ and WT mice (*n* = 5–7 mice per group). (E) Plasma vitamin E concentration in human subjects (patients, *n* = 3; parents, *n* = 4; normal individuals, *n* = 19). (F) Plasma vitamin E concentration in human subjects after oral ingestion of 600 mg of d-*a*-tocopherol (patient, *n* = 1; parents, *n* = 2; normal individuals, *n* = 8). (G, H) Time to cross the beam and number of foot slip errors by beam walk test in the indicated mice at different ages (*n* = 8 mice per group). (I–K) Latency to fall measured by rotarod test in the indicated mice at different ages (*n* = 8–9 mice per group). (L) Confocal images of MBP (red) immunostaining in pons region in the indicated mice. Nuclei were counterstained with DAPI. (M, N) Percentage of field area positive for MBP (M) and quantification of the relative MBP immunodensity (*N*) in pons region of the indicated mice (*n* = 4–6 mice per group, 2–3 fields per mouse). Scale bars, 50 μm in (L). Means (± SEM) are plotted with each symbol representing an individual animal in (A–D). Means (± SEM) are plotted with each symbol representing an individual data point in (M, N). Means (± SEM) are plotted with each symbol representing an individual person in (E). Means with bars indicating SEM are plotted in (F–K). *P* values were calculated with two-tailed Student’s *t*-test in (A–D), one-way ANOVA with the Dunnett correction for multiple tests in (E–N), and two-way ANOVA with the Bonferroni correction for multiple tests in (F–K). NS indicates no significant difference (*P* > 0.05). *P < 0.05, **P < 0.01, ***P < 0.001, ****P < 0.0001.

To evaluate whether inhibiting FXR with IVM treatment could improve the defects of lipid absorption and neuromotor function, we administered IVM to *Scarb2*^−/−^ mice via intragastric administration every two days starting from 1.5 months of age. A 3.5-month-long course of IVM treatment resulted in significant weight gain in *Scarb2*^−/−^ mice (Fig. S5A), with no effect on food intake (Fig. S5B). However, IVM treatment did reduce energy expenditure in 5-month-old *Scarb2*^−/−^ mice (Fig. S5C and S5D). To confirm the inhibition of FXR signaling in the intestine due to IVM treatment, we assessed the mRNA levels of relevant genes. IVM treatment significantly decreased the mRNA levels of FGF15, SHP and Ibabp (Fig. 6E). Furthermore, IVM treatment increased the mRNA levels of key genes involved in fat absorption in jejunum (Fig. 6F) and bile acids synthesis in liver (Fig. 6G). The fat absorption capacity was subsequently evaluated in IVM-treated and control animals. IVM treatment did not alter dietary fat absorption in WT mice (Fig. 6H and 6I), but elevated circulating TG levels in *Scarb2*^−/−^ mice, both in the presence and absence of tyloxapol (Fig. 6H and 6I). Notably, the enhancement of TG levels was more pronounced in the presence of tyloxapol, suggesting accelerated TG uptake into tissues following intestinal absorption in *Scarb2*^−/−^ mice. The neuromotor functions were further analyzed in IVM-treated *Scarb2*^−/−^ mice. Locomotor activity was assessed using the beam walk test, revealing a significant improvement in the performance of IVM-treated *Scarb2*^−/−^ mice compared to those treated with the vehicle (Fig. 6J and 6K). Additionally, mice were subjected to the accelerating rotarod performance test at different ages, showing that IVM-treated *Scarb2*^−/−^ mice outperformed their vehicle-treated counterparts (Fig. 6L–O). To assess the impact of IVM supplementation on neuropathy, we examined astrogliosis in the pons region of 5-month-old mice. Vehicle-treated *Scarb2*^*−/−*^ mice exhibited significant astrogliosis, as indicated by GFAP staining, in the pons region compared to WT mice of the same age (Fig. S8A and S8B). However, astrogliosis was notably lessened in IVM-supplemented *Scarb2*^*−/−*^ mice relative to their vehicle-treated counterparts (Fig. S5E and S5F). Myelin basic protein (MBP), a key component of the myelin sheath of oligodendrocytes in the central nervous system, was assessed to evaluate myelination. MBP staining in the pons region of 5-month-old mice (Fig. S5G) was quantified by measuring both the MBP-positive area and MBP immunodensity. This quantification revealed a significant decrease in MBP-positive area and immunodensity in *Scarb2*^*−/−*^ mice compared to WT mice (Fig. 7L and 7N). However, these reductions in MBP-positive area and immunodensity were significantly mitigated by IVM treatment (Fig. S5H and S5I). Collectively, these data indicate that 5-month-old *Scarb2*^−/−^ mice exhibit hyperactivated FXR signaling in intestine and liver, resulting in reduced BA synthesis and impaired intestinal epithelium renewal. Blocking FXR signaling alleviates defects in fat absorption, intestinal renewal and neuromotor functions in *Scarb2*^−/−^ mice.

### Deficiency of vitamin E in *Scarb2*^−/−^ mice

Fat-soluble vitamins are absorbed along with dietary fat. Fat malabsorption is often associated with vitamin deficiency. We measured the level of fat-soluble vitamins in plasma in 5-month-old animals. *Scarb2* knockout led to a significant decrease in the plasma levels of vitamin E (Fig. 7A) and other fat-soluble vitamins (Fig. S6A–D). Alpha-tocopherol is the most biologically active form of vitamin E, and vitamin E refers to alpha-tocopherol in the text hereafter. In contrast, the plasma levels of water-soluble vitamins, such as vitamins B3, B12 and C, were comparable between WT and *Scarb2*^−/−^ mice (Fig. S6E–G). This indicates a specific deficit in fat-soluble vitamins.

Since vitamin E is known for its neuroprotective effect, we next determined the abundance of vitamin E in tissues. The vitamin E level was decreased in brain homogenates of *Scarb2*^−/−^ mice (Fig. 7B). Vitamin E is stored in liver and adipose tissue. We found that the level of vitamin E was dramatically reduced in liver tissues in 5-month-old *Scarb2*^−/−^ mice relative to WT mice (Fig. 7C). In fat tissue, the reduction of vitamin E was milder but still significant in *Scarb2*^−/−^ mice (Fig. 7D).

To determine whether human patients with *SCARB2* mutation have a similar reduction in vitamin E level, we obtained blood samples from three patients with *SCARB2* mutations. Two of the patients were previously reported (He et al., 2014). By genomic DNA sequencing, we found that the third patient has a homozygous nonsense mutation, c.1270C > T (p.R424X), in the coding region (exon 11) of the *SCARB2* gene (Fig. S7). The plasma concentration of vitamin E was significantly lower in blood samples from the three patients, compared to their parents and normal controls (Fig. 7E). We further performed a vitamin E tolerance test in one living patient, along with the parents and healthy controls. After a dietary challenge of vitamin E, the plasma concentration of vitamin E in the patient’s parents and healthy controls rose during the following 24 h, reaching a peak at 8 h. However, the plasma concentration of vitamin E failed to increase significantly in the patient (Fig. 7F). Thus, the patient displayed a profound defect in absorbing dietary vitamin E.

### Supplementation of vitamin E ameliorates the neuromotor deficit and neuropathy of *Scarb2*^−/−^ mice

Vitamin E deficiency has been associated with ataxia, and supplementation of vitamin E can rescue the neuromotor deficit in mice lacking alpha-tocopherol transfer protein (Yokota et al., 2001). We investigated whether vitamin E supplementation could ameliorate some of neuromotor deficits in *Scarb2*^−/−^ mice. Locomotor activity was assessed with the beam walk test. Based on the crossing time and the number of foot slip errors, there was a significant improvement in the performance of *Scarb2*^−/−^ mice treated with vitamin E compared to *Scarb2*^−/−^ mice treated with vehicle (Fig. 7G and 7H). Mice were also subjected to the accelerating rotarod performance test at different ages. Vitamin E-supplemented *Scarb2*^−/−^ mice outperformed the vehicle-treated *Scarb2*^−/−^ mice at late time points (4.5 and 5.5 months of age), but not the early time point (3.5 months of age) (Fig. 7I–K).

To investigate the effects of vitamin E supplementation on neuropathy in *Scarb2*^−/−^ mice, we evaluated astrogliosis in the pons region in 5-month-old mice. Astrogliosis was reduced in vitamin E-supplemented *Scarb2*^−/−^ mice compared to vehicle-treated *Scarb2*^−/−^ mice (Fig. S8A and S8B). We also compared the number of TUNEL^+^ cells in the vitamin E-supplemented and control groups. Vitamin E treatment significantly reduced the number of TUNEL^+^ cells in pons in *Scarb2*^−/−^ mice (Fig. S8C and S8D). Among the TUNEL^+^ cells, the number of neurons, labeled by tubulin III, was significantly reduced in pons in vitamin E-treated animals compared to the vehicle-treated *Scarb2*^−/−^ mice (Fig. S8E and S8F). We also evaluated myelination by staining MBP in pons region in 5-month-old mice (Fig. 7L). The quantification showed a marked reduction of MBP-positive area in *Scarb2*^−/−^ mice compared to WT mice, and the reduction of MBP-positive area was significantly ameliorated by vitamin E treatment (Fig. 7M). Quantification of MBP immunodensity showed a marked reduction in *Scarb2*^−/−^ mice compared to WT mice (Fig. 7N). Vitamin E supplementation alleviated the reduction of MBP immunodensity in *Scarb2*^−/−^ mice (Fig. 7N). Thus, vitamin E supplementation ameliorates some of the neuromotor deficits and also the neuropathy observed in *Scarb2*^−/−^ mice.

## Discussion

Our data demonstrate that Scarb2 deficiency causes marked dysbiosis and alters the bile acid pool, resulting in abnormal activation of FXR in small intestine. Abnormal activation of FXR leads to impaired epithelium renewal and reduced synthesis of bile acids in liver, which decreases dietary fat absorption in *Scarb2*^−/−^ mice in an age-dependent manner. We also show that blocking FXR to restore fat absorption or addressing the vitamin E deficiency associated with dietary fat malabsorption ameliorates the neurodegeneration associated with Scarb2 deficiency.

Our data suggest that Scarb2 deficiency is linked to significant dysbiosis. We observed a notable increase in the abundance of BSH-active bacteria, specifically bacteria from the *Lactobacillus* and *Bifidobacterium* genera, in 5-month-old *Scarb2*^−/−^ mice. Additionally, *Scarb2*^−/−^ mice exhibited significantly higher BSH activity. BSH-active bacteria are typically considered beneficial in mitigating metabolic disorders. However, excessive BSH activity could potentially lead to adverse effects on the host, such as lipid malabsorption (Choi et al., 2015). Interestingly, previous studies have reported elevated abundance of the *Bifidobacterium* and *Lactobacillus* genera in various PD patient groups (Li et al., 2023). Therefore, it is essential to investigate whether the increased abundance of *Bifidobacterium* and *Lactobacillus* genera is associated with abnormal weight or disease progression in PD patients.

BSH, as a key enzyme in secondary bile acid metabolism, plays a major role in modulating microbiome-BA-FXR crosstalk. Gut bacteria modulate FXR activity by altering BAs. For instance, unconjugating tauro-alpha- and tauro-beta-MCA can alleviate FXR inhibition (Sayin et al., 2013). FXR activation negatively regulates BA synthesis in the liver either directly or through the action of intestine-derived FGF15 (Wahlström et al., 2016). FGF15 expression was significantly elevated in *Scarb2*^−/−^ mice. Key genes involved in BA synthesis were downregulated in liver, which is consistent with the reduced abundance of BAs in gallbladder in 5-month-old *Scarb2*^−/−^ mice. In the small intestine, the genes involved in fatty acid uptake were downregulated in 5-month-old *Scarb2*^−/−^ mice, consistent with the finding that FXR activation limits intestinal lipid uptake (Clifford et al., 2021). SHP was also upregulated in small intestine in 5-month-old *Scarb2*^−/−^ mice. A previous study has indicated intestinal SHP as a potential global repressor of intestinal lipid synthesis/absorption and bile acid recycling genes (Seok et al., 2022). Inhibiting FXR with IVM largely rescued the defect in dietary fat absorption seen in *Scarb2*^−/−^ mice. Thus, we postulate that higher microbial BSH activity and hyperactivation of FXR may underlie the lipid malabsorption in 5-month-old *Scarb2*^−/−^ mice.

Epithelium renewal was severely delayed in 5-month-old, but not in 2-month-old, *Scarb2*^−/−^ mice. Inhibition of FXR rescued the impaired epithelium turnover *in vitro* and *in vivo*, which indicates that FXR hyperactivation in 5-month-old *Scarb2*^−/−^ mice underlies the epithelium turnover defect. Our observation is consistent with the previous reports showing that BAs can regulate intestinal stem cell proliferation by agonizing or antagonizing FXR activity (Dossa et al., 2016; Fu et al., 2019). Notably, GI dysfunction and dysbiosis are observed in a number of neurodegenerative diseases (Marizzoni et al., 2017; Romano et al., 2021; Warnecke et al., 2022). Investigations are warranted to determine whether the intestinal turnover is implicated in a similar manner in other disease models.

FXR orchestrates complex interactions in the gut-liver axis, mediating the influence of microbiota on bile acid synthesis (Sayin et al., 2013). Mice with intestine-specific FXR deletion exhibit resistance to diet-induced obesity, highlighting the receptor’s role in energy balance and metabolic regulation (Jiang et al., 2015b; Li et al., 2013). Additionally, activation of intestinal FXR has been associated with increased energy expenditure in brown adipose tissue, demonstrating a protective mechanism against metabolic dysregulation induced by a high-fat diet (Fang et al., 2015). These findings reveal the dual protective roles of intestinal FXR, emphasizing the intricate relationship among diet, bile acid composition, and FXR signaling in maintaining metabolic health. 5-month-old *Scarb2*^−/−^ mice had significantly higher energy expenditure than controls, while younger mice had comparable energy expenditure with controls. Zou et al. reported that 4-month-old *Scarb2*^−/−^ mice had higher energy expenditure with enhanced glycolysis in adipocytes (Zou et al., 2022). Our data indicate that in addition to the adipocyte-intrinsic function of Scarb2, Scarb2 deficiency alters BA-FXR signaling in an age-dependent manner, which might lead to an overall change in host metabolism. Indeed, inhibiting FXR with IVM treatment reduced energy expenditure in *Scarb2*^−/−^ mice, which is consistent with the previous studies on FXR signaling in host metabolism (van Zutphen et al., 2019).

Chronic fat malabsorption often results in deficiency of multiple fat-soluble vitamins, including vitamins A, D, E and K. We focused our study on correcting the deficiency of vitamin E, because vitamin E deficiency is known for its role in ataxia, which is one of the clinical symptoms present in patients with *SCARB2* mutations. Supplementation of vitamin E ameliorated some neuromotor impairments in *Scarb2*^−/−^ mice, evaluated by beam walk and rotarod tests. Also, supplementation of vitamin E reduced the extent of astrogliosis observed in *Scarb2*^−/−^ mice. In previous studies, vitamin E supplementation has been shown to be generally neuroprotective in conditions associated with neuronal damage (Santander et al., 2017; Schirinzi et al., 2019; Yokota et al., 2001). Our study does not provide direct evidence that vitamin E supplementation offers specific benefits to *Scarb2*^−/−^ mice rather than a general neuroprotective role. Vitamin E deficiency was also observed in patients with *SCARB2* mutations. One patient who was examined showed a profound defect in absorbing dietary vitamin E. The data from patients are consistent with our data from *Scarb2*^−/−^ mice. More patients should be included in future studies.

Lipid malabsorption does not generally occur in GD and PD. However, plasma levels of essential fatty acids, such as alpha-linolenic acid, linoleic acid, and arachidonic acid, were lower in patients with PD (Yoo et al., 2021), indicating altered lipid metabolism. Compared to GD and PD, PME-4 is a rather rapidly progressive neurodegeneration. The defect in fat absorbing might become quite exacerbated in the context of complete loss of Scarb2. Our study indicates that nutritional status should be carefully examined in more SCARB2-associated neurodegeneration.

In summary, we report that Scarb2 deficiency leads to gut dysbiosis and dysregulated BA-FXR crosstalk, which impairs lipid absorption and epithelium renewal in the host. Inhibiting FXR signaling or supplementation of vitamin E in Scarb2-deficient mice was found to mitigate neurodegeneration. Thus, our study identifies a key role of abnormal BA-FXR crosstalk underlying the neurodegeneration-associated gut dysbiosis in the host.

## Materials and methods

### Antibodies and reagents

#### Antibodies

Anti-GFAP, anti-Myelin Basic Protein, anti-SOX9 were obtained from Abcam; anti-Tubulin β-3 from Biolegend; anti-Ki67 from Invitrogen; anti-β-catenin from Santa Cruz; anti-Rabbit Alexa Fluor 555 tagged, anti-Rabbit Alexa Fluor 488 tagged, anti-Mouse Alexa Fluor 555 tagged from Thermo Fisher Scientific.

#### Reagents

Normal goat serum blocking reagent were obtained from Boster Biotech; DAPI from Novon; Fluoromount-G from Southern Biotech; Eosin solution from ZSGB-BIO; Olive oil from Sangon Biotech; Oil Red O from BBI Life Science; EDU from Beyotime; Ninhydrine from Macklin; GW4064 from Abmole; Ivermectin from MCE; Tauro-β-Muricholic Acid Sodium Salt from Yuanye; Corning Matrigel Matrix from Corning; Advanced DMEM/F-12 from Gibco; HEPES from Gibco; B27 and N2 from Thermo Fisher Scientific; Recombinant Murine EGF, Recombinant Murine Wnt-3a, and Recombinant Murine Noggin from Peprotech; Vitamin E soft capsules from Zhejiang Medicine. All other reagents were from Sigma-Aldrich unless indicated otherwise.

### Human subjects

The human subjects included 3 PME-4 patients. Two of the patients were sisters in the same family (aged 30 and 37 when samples were taken) (He et al., 2014). The plasma samples were stored at −80°C. The third patient was a female aged 30. The ages of the third patient’s parents were 56 and 58. Genomic DNA analysis showed that the third patient had a homozygous nonsense mutation, c.1270C > T (p.R424X), in the coding region (exon 11) of SCARB2:NM_005506. The parents were heterozygous for the mutation. The control group consisted of 19 normal adults (mean age 28 ± 2.34 years; 10 women, 9 men). Blood was drawn from all subjects.

### Oral vitamin E tolerance test

Intestinal absorption of vitamin E was assessed in one patient (aged 30, woman), her parents (aged 56 and 58), and normal adults (aged 26.5 ± 02.14, 4 women, 4 men). The oral vitamin E tolerance test was performed as described (Sokol et al., 1983). After an 8 h overnight fast, the subjects ingested 20 mL milk containing 600 mg of liquid emulsified d-*a-*tocopherol (Zhejiang Medicine). Normal diets and prior medications (excluding oral vitamin E) were resumed 1 h later. After the oral dose, plasma samples were obtained at 0, 4, 8, 12, and 24 h. Plasma vitamin E concentration was determined by LC–MS.

### Mice

*Scarb2* ^−/−^ mice (C57BL/6J background) were a gift from professor Liguo Zhang from the Institute of Biophysics, Chinese Academy of Sciences. *Scarb2*^−/−^ mice were generated using the CRISPR/Cas9 method which delete 14 nucleotides in exon 1 of the Scarb2 gene. Male mice in 2–6-month-old were used in this study unless noted otherwise. All specific pathogen-free (SPF) mice were housed at 2–5 animals per cage in the AAALAC (Association for Assessment and Accreditation of Laboratory Animal Care) accredited barrier facility in Tsinghua University and bred in a 12/12 h light/dark cycle with ad libitum access to food and water at a controlled temperature (23°C ± 2°C). All mice fed a regular diet.

### Mouse intestinal mesenchymal stromal cell culture

Mouse intestinal mesenchymal stromal cell isolation and culture have been reported previously (Koliaraki et al., 2015). Briefly, 5–8 cm small intestine were taken from 8-week-old euthanized mice, cut into 1 mm pieces and washed with HBSS. Tissues were then incubated in HBSS with 5 mmol/L EDTA and 1 mmol/L DTT for 20 min at 37°C to remove the intestinal epithelium. Intestinal pieces were then digested in DMEM containing 0.5 mg/mL collagenase IV and 0.5 mg/mL dispase II for 30 min at 37°C. Digested supernatants were centrifuged and cells were cultured in DMEM medium with 10% heat-inactivated FBS, 1% nonessential amino acids, 100 U/mL penicillin, 100 µg/mL streptomycin, and 1% L-glutamine.

### Mouse intestinal organoid culture

Mouse intestinal organoid culture has been described in previous reports (Zhang et al., 2015). A 5-cm-long section of distal jejunum from euthanized mice was opened longitudinally and rinsed with cold PBS. The tissue was then washed vigorously in 20 mL cold PBS in a 50-mL tube for 1 min and rinsed again in cold PBS. The tissue was then chopped into pieces smaller than 3–4 mm and incubated at 37°C for 20 min with 2 mL 0.1% collagenase solution. Tissue sections were pipetted up and down every 7 min during the incubation. Tissue sections were then transferred to another 2 mL of fresh collagenase solution for 20 min, during which the digestion reaction was pipetted up and down every 7 min and monitored for the release of crypts. Digestion was stopped by dilution with 20 mL cold PBS when around 70% of the crypts had been released, and the digests were filtered through a 70-μm cell strainer. The released crypts were pelleted by centrifugation at 100 ×*g* for 5 min. Pelleted crypts (~500) were then mixed with 50 μL Matrigel and plated in 24-well plates. After Matrigel polymerization, 500 μL growth medium was added to each well. Organoids were retrieved from the Matrigel, mechanically dissociated and a total of 100 crypts were mixed with 1 × 10^4^ mouse intestinal mesenchymal cells and plated into fresh Matrigel in 96-well plates after two days and treated with GW4046 (10 μmol/L) or T-β-MCA (100 μmol/L). Growth medium was changed every 3 or 4 day (d). Culture medium contained Advanced DMEM/F12 supplemented with L-glutamine, 1% Pen-Strep, 10 μmol/L HEPES, N2 supplement 1:100, B27 supplement 1:50, 50 ng/mL epidermal growth factor, 500 ng/mL R-spondin 1, 100 ng/mL Noggin and 10 ng/mL Wnt-3a.

### Vitamin E supplementation

Water-soluble vitamin E was prepared by dissolving d-*α*-tocopherol polyethylene glycol 1000 succinate (Sigma-Aldrich) in saline at a concentration of 4% (*w*/*v*). Mice were treated with 200 μL vitamin E solution every 3 days by intravenous injection via tail vein. Control mice were treated with 200 μL saline every 3 days by tail vein injection. Mice started to receive the vitamin E (or vehicle) treatment regimen when they were 4 weeks old.

### Ivermectin treatment

To evaluate epithelial renewal using EdU-labeling, 4-month-old *Scarb2*^−/−^ mice received an oral gavage of Ivermectin (3 mg/kg, dissolved in 1% DMSO, 4% PEG300, 5% Tween-80, and 90% saline) or vesicle once a day for a duration of 14 days.

For the 3.5-month-long treatment, 1.5-month-old *Scarb2*^−/−^ mice were administered Ivermectin (3 mg/kg, dissolved in 1% DMSO, 4% PEG300, 5% Tween-80, and 90% saline) or vesicle once every two day. Throughout the treatment period, assessments of motor functions, fat absorption, and intestinal renewal were conducted on the mice.

### Fecal microbiota transplantation (FMT)

Three-month-old male mice were treated with ABX for 1 week and then rest for 3 days before fecal microbiota transplantation. ABX mice were prepared according to the published procedure (Hill et al., 2010). In brief, mice were subjected to oral gavage daily for 7 days with 300 μL of autoclaved water or autoclaved water supplemented with ampicillin (1 mg/mL), gentamicin (1 mg/mL), metronidazole (1 mg/mL), neomycin (1 mg/mL), and vancomycin (0.5 mg/mL). Mouse fecal sample were collected in dry ice and stored at −80°C until DNA extraction. Feces were weighted and DNA from intestinal microbiota was isolated with the Qiagen stool isolation kit according to the manufacturer’s instructions. Total fecal bacterial abundance was quantified by real-time qPCR analysis of total 16s rRNA copy numbers normalized to feces weight (340F, 5ʹ-ACTCCTACGGGAGGCAGCAGT-3ʹ; 514R, 5ʹ-ATTACCGCGGCTGCTGGC-3ʹ). Five-month-old *Scarb2*^−/−^ and WT mice cecum microbiota were collected and dissolve in cold PBS for gavage. *Scarb2*^−/−^ or WT mice cecum microbiota suspension were orally administrated to ABX treated mice once a day for 2 weeks.

### Behavioral tests

All behavioral tests of mice were conducted during the light cycle by a researcher blinded to the genotype of the mice. Repeated tests were carried out at the same time of the day.

### Wire hang test

The wire hang test was performed to evaluate the balance and grip strength of mice (Zhu et al., 2016). Briefly, the animal was hung upside down on a wire mesh (12 mm × 12 mm grids) placed 30 cm above a mouse cage. The time until the mouse fell off the wire mesh into the cage was recorded. If the mouse did not fall within 300 s, the mouse was removed from the wire mesh and assigned a maximum score of 300 s.

### Beam walk test

The time to cross the center 70 cm of a 100 cm beam and the number of foot slip errors were recorded. A beam of 10 mm in width and 5 mm in thickness was mounted atop poles (15 cm above the table top) with a housing box containing the animal’s home cage nest material (the goal box) at the far end. A camera was used to record the beam crossing performance. The test includes one day training and two days testing. Before every trial, the animal was habituated in the goal box for 3 min. On the training day, the animal was encouraged to cross the beam by nudging and tail pinching until it crossed the beam three times without stopping and turning around. On the testing days, three trials were performed for each animal. Trials in which the animal stopped or turned around were repeated. The time to cross the beam and the number of foot slips of the three trials were averaged for each animal (Southwell et al., 2009).

### Rotarod test

The rotarod test was performed using an accelerating rotarod system (YLS-4C, Jinan Yiyan Technology Development). The test included 1 day of training and 2 days of testing. Four trials were performed per day. During the training day, the mouse was placed on 30 mm diameter rods and was trained at 10 rpm for 300 s. During the testing days, the rotarod accelerated from 5 rpm to 30 rpm within 90 s and the time until the mouse fell off the rod was recorded. If the mouse did not fall within 300 s, the mouse was removed from the rotarod and assigned a maximum score of 300 s. Rotarod trials were performed with a 20 min inter-trial interval. The trials on the second day of testing were scored (Southwell et al., 2009).

### Determination of serum FGF15 content

Mice were fasted for 6 h at day time and serum was collected via orbital sinus bleeding from anaesthetized mice. FGF15 concentrations were measured via ELISA (ELK Biotechnology), following the manufacturer’s instructions.

### Body composition analysis

The body composition of animals was analyzed by an EchoMRI body composition analyzer system (EchoMRI 100 system, EchoMRI Medical System, Houston, TX). Briefly, conscious mice were weighed and then placed in a thin-walled plastic cylinder with a cylindrical plastic insert added to limit movement of mice. Mice were briefly subjected to a low intensity electromagnetic field. Fat mass, lean mass, free water and total water were measured.

### Hematoxylin and eosin staining

Tissues (fat and intestine) were collected from euthanized mice and fixed in 4% PFA at 4°C overnight. Formalin-fixed and paraffin-embedded tissues were stained with hematoxylin & eosin (H&E). Briefly, tissue slices (6 μm in thickness) were mounted on positively charged glass and dewaxed. Slides were stained by hematoxylin (Sigma-Aldrich) and eosin (ZSGB-BIO) and then mounted. Images were captured with a 3DHISTECH SCAN II scanner (3DHISTECH, Budapest, Hungary).

### Serum and tissue triglyceride and NEFA measurement

Mice were fasted overnight and blood samples were collected into tubes through orbital sinus bleeding of anaesthetized mice. Serum samples were prepared and serum triglyceride was measured by commercially available TG kit (BIOSINO).

Tissues (liver and duodenum) were collected from euthanized mice. Fifty milligrams tissues were homogenized in 500 µL cold dichloromethane/methanol (2:1, *v*/*v*). 125 µL ddH_2_O was added into the tissue lysate and the mixture was centrifugated at 12,000 rpm for 15 min. A total of 100 µL organic solvent was transferred to a new tube and dried under stream of nitrogen. The pellets were dissolved by 200 µL ethanol and the concentration of triglyceride and NEFA were determined by commercially available TG kit (BIOSINO).

### Oil red O staining

Tissues were collected from euthanized mice and fixed in 4% PFA at 4°C for 12 h and then incubated in 30% sucrose for 12 h at 4°C. Fixed tissues were embedded in Tissue-Tek O.C.T. Compound (Sakura Finetek, Torrance, CA, USA). Serial sections (12 μm in thickness) were made and stained with Oil Red O (BBI Life Science) working solution (0.5% Oil Red O in isopropanol: ddH_2_O = 3:2, *v*/*v*) for 10 min, counter-stained with hematoxylin (Sigma-Aldrich). Images were captured with a 3DHISTECH SCAN II scanner (3DHISTECH, Budapest, Hungary).

For oil gavage and the duodenum staining, animals were fasted overnight and then administered with an oral gavage of olive oil (10 µL/g body weight, Sangon Biotech). Duodenum sections were collected 2 h post-gavage from euthanized mice.

For Oil Red O staining of liver tissues, mice were not fasted. Livers were collected from euthanized mice without oil gavage.

### Metabolic cages

Mice were individually housed for 2 days before metabolic analysis. Indirect calorimetric including oxygen (O_2_) consumption, carbon dioxide (CO_2_) expiration, energy expenditure (EE), RER, and food consumption were assessed for 4 days using the Phenomaster system (TSE Systems GmbH, Bad Homburg, Germany). Data analysis and plotting for metabolic studies were performed in the R programming language with CalR, a custom package for analysis of indirect calorimetry using analysis of covariance with a graphical user interface (Mina et al., 2018).

### Plasma long-chain fatty acid extraction and analysis

Mice were fasted overnight. Blood was collected via orbital sinus bleeding from anaesthetized mice. Plasma long-chain fatty acid extraction was described previously (Kamphorst et al., 2011). The plasma was prepared from blood by centrifugation at 3,000 ×*g* for 10 min at 4°C. A total of 100 μL plasma was mixed with 400 μL ice-cold extraction solution (methanol:dichloromethane = 1:2, *v*/*v*) followed by vigorous vertexing for 1 min and centrifugation at 12,000 rpm for 20 min. The dichloromethane layer was transferred to a new tube followed by drying under nitrogen flow. Dried pellets were resuspended using 1 mL working solution (methanol: ddH_2_O = 9:1, *v*/*v*, containing 0.3 mol/L KOH) and saponified in an 80°C water bath for 1 h. Then, the mixtures were acidified by addition of 100 μL of formic acid and extracted with 1 mL of hexane. The hexane layer was collected to a new tube, dried under nitrogen flow and resuspended in 120 μL of dichloromethane/methanol buffer (1:1, *v*/*v*) prior to LC-MS analysis.

Long-chain fatty acids were analyzed using ultra-high pressure liquid chromatography (Ultimate 3000^TM^ UHPLC system, Thermo Fisher, USA) coupled to a quadrupole orbitrap mass spectrometer (Q-Exactive HF^TM^ Orbitrap MS system, Thermo Fisher, USA) equipped with a heated electrospray ionization (HESI) probe. Lipid extracts were separated by a CORTECS C_18_ column (100 mm × 2.1 mm, 2.7 μm; Waters, USA) at a flow rate of 250 μL/min. The LC elution gradient using a mobile phase of solvent A (acetonitrile: ddH_2_O = 6:4, *v*/*v*, containing 10 mmol/L ammonium acetate) and solvent B (isopropanol: acetonitrile = 9:1, *v*/*v*) was optimized as follows: 0–2.5 min, 30% B; 2.5–8 min, 30% B to 50% B; 8–10 min, 50% B to 98% B; 10–15 min, 98% B; 15–15.1 min, 98% B to 30% B; and 15.1–18 min, 30% B. The column temperature and sample tray were maintained at 40°C and 10°C, respectively. The eluted lipids were inlet to high-resolution MS by negative electrospray ionization mode with an ion-spray voltage of 3,000 V, capillary temperature of 320°C, heater temperature of 300°C, sheath gas flow rate of 35 arb and auxiliary gas flow rate of 10 arb. The MS analysis was processed with a mass range of *m*/*z* 150–600 and a resolution of 70,000 FWHM. TraceFinder (Thermo Scientific, USA) software was employed to identify lipids with an endogenous MS database by accurate masses. The following six individual fatty acids were measured in our study: stearic acid (18:0), oleic acid (18:1), linoleic acid (18:2), linolenic acid (18:3), palmitic acid (16:0), and palmitoleic acid (16:1).

### Postprandial triglyceridemic response

Mice were fasted overnight and given an oral gavage of olive oil (10 µL/g body weight, Sangon Biotech). Blood was collected at five time points up to 4 h post-gavage via the tail vein. No food was available throughout the collection. Serum samples were prepared from the blood, and serum triglyceride concentration was determined by commercially available TG kit (BIOSINO).

### Intestinal TG secretion

Mice were fasted overnight and then intraperitoneally injected with tyloxapol (500 mg/kg body weight, Sigma-Aldrich) to inhibit lipoprotein lipase activity in the circulation. After 40 min, mice were administered with an oral gavage of olive oil (10 µL/g body weight, Sangon Biotech). Blood was collected at five time points up to 4 h post-gavage via tail vein. Food was not available during the collection. Serum samples were prepared from the blood. Triglyceride in the serum was detected by commercially available kits (BIOSINO).

### Lipoprotein analysis by fast performance liquid chromatography (FPLC)

Lipoprotein analysis by fast performance liquid chromatography has been reported previously (Kim et al., 2020). Briefly, mice were given an oral gavage of olive oil (10 µL/g body weight). Two hours after gavage, plasma was collected via orbital sinus bleeding from anaesthetized mice. Plasma (200 µL) was subjected to gel filtration with FPLC for lipoprotein analyses. A GE Healthcare ÄKTA Explorer equipped with a Superose 6 10/300 GL column (GE Healthcare Bio-Science AB, Björkgatan, Uppsala, Sweden) was employed with 10 mmol/L sodium phosphate, 0.15 mol/L sodium chloride, and 0.01% (*w*/*v*) sodium EDTA, pH 7.2 at 4°C. The FPLC system was run with a constant flow of 300 μL/min, and fractions were collected after 7 min with 400 μL per fraction. The concentrations of triglyceride in fractions 1–35 were determined according to the manufacturer’s instruction using commercially available kits (BIOSINO).

### Lipase activity

Intestinal mucosa samples were scraped from the same length of intestine and then suspended in 200 µL cold PBS. The intestinal mucosa suspension was centrifugated for 10 min at 15,000 ×*g* and the supernatant was assayed for lipase activity using commercially available kits (BIOSINO). Blood was collected via orbital sinus bleeding from anaesthetized mice. Plasma samples were prepared, and plasma lipase activity was determined using commercially available kits (BIOSINO).

### Bile acid extraction and measurement

The gallbladders of euthanized mice were collected and punctured with a 25 G needle, releasing their contents. The contents of the gallbladders were diluted in 75% ethanol for further analysis. For extraction of bile acids from tissues, 100 mg of liver tissue was homogenized in 1 mL 75% ethanol and whole intestine was homogenized in 10 mL 75% ethanol. The homogenate was incubated at 50°C for 2 h and centrifuged at 12,000 ×*g* for 10 min. The supernatant was used for measurement of bile acids. Blood was collected via orbital sinus bleeding from anaesthetized mice and serum samples were prepared for bile acid measurement. The concentrations of total bile acids were measured by commercially available kits (BIOSINO).

### Microbiota DNA extraction and analyses

Mouse fecal sample were collected in dry ice and stored at −80°C until DNA extraction. Microbiota DNA extraction and analysis have been described in a previous publication (Zhang et al., 2015). Feces were weighted and DNA from intestinal microbiota was isolated with the Qiagen stool isolation kit according to the manufacturer’s instructions. DNA concentration was measured by NanoDrop and molecular size was estimated by agarose gel electrophoresis. Total fecal bacterial abundance was quantified by real-time qPCR analysis of total 16s rRNA copy numbers normalized to feces weight (340F, 5ʹ-ACTCCTACGGGAGGCAGCAGT-3ʹ; 514R, 5ʹ-ATTACCGCGGCTGCTGGC-3ʹ). Specific primers were used to determine the relative abundance of selected bacteria by real-time PCR, including *Bifidobacterium* (F: 5ʹ-GGGTGGTAATGCCGGATG-3ʹ; R: 5ʹ-CCACCGTTACACCGGGAA-3ʹ) and *Lactobacillus* (F: 5ʹ-AGCAGTAGGGAATCTTCCA-3ʹ; R: 5ʹ-CACCGCTACACATGGAG-3ʹ). The results were normalized to total bacterial 16s rRNA gene copy numbers.

### 16s rRNA gene sequencing

The following procedures were carried out by Majorbio BioPharm Technology Co., Ltd. (Shanghai, China). Universal primers 338F and 806R were used for PCR amplification of the V3–V4 hypervariable regions of 16S rRNA genes (338F, 5ʹ-ACTCCTACGGGAGGCAGCA-3ʹ; 806R, 5ʹ-GGACTACHVGGGTWTCTAAT-3ʹ). Sequencing was done on an Illumina MiSeq PE300 (Illumina, San Diego, USA). The QIIME (1.9.1) software package was used to analyze the sequence data. The RDP Classifier algorithm against the Silva 16S rRNA database (silva 128/16s bacteria) using a confidence threshold of 70% was used to analyze the taxonomy of each 16S rRNA gene sequence. Operational taxonomic units (OTUs) were clustered using UPARSE version 7.1 at a 97% similarity cutoff, and chimeric sequences were identified and removed. The alpha-diversity and statistical data were calculated based on the Shannon and Chao indexes. The beta-diversity was measured using principal coordinate analysis (PCoA) of Bray-Curtis dissimilarity. 16S rRNA gene sequencing data have been submitted to NCBI Sequence Read Archive (SRA) with the accession number of PRJNA979172.

### Cecum BSH activity

Cecum bile salt hydrolase activity based on the generation of taurine from taurodeoxycholic acid, was measured by a previously described method with several modifications (Ahmadi et al., 2020). Briefly, 200 mg cecal contents were suspended in 2 mL 0.1 mol/L sodium phosphate buffer (pH = 6.0) and vortexed. The mixture was settled on ice for 20 min to remove the precipitate. Next, 178 µL supernatant was mixed with 2 µL 1 mol/L DTT and 20 µL 100 mmol/L taurodeoxycholic acid (sodium salt) and incubated at 37°C overnight. The reaction was stopped by adding 50 µL 15% (*w*/*v*) trichloroacetic acid. Then, the sample was centrifuged at 12,000 ×*g* for 5 min, and 100 µL supernatant was mixed with 400 µL 1% (*w*/*v*) ninhydrin and 100 µL 0.5 mol/L pH = 5.5 sodium citrate buffer. The mixture was incubated at 95°C for 10 min. The absorbance at 570 nm was determined using taurine as the standard.

### Serum bile acid analyses

Serum was collected via orbital sinus bleeding from anaesthetized mice. 100 μL serum was mixed with 400 μL ice-cold methanol. The mixture was vortexed for 5 min and placed on ice for 20 min, followed by centrifugation at 12,000 ×*g* for 10 min at 4°C. The supernatant was evaporated and resuspended in 50 μL methanol for LC-MS analysis.

Bile acids were analyzed using ultra-high pressure liquid chromatography (Ultimate 3000^TM^ UHPLC system, Thermo Fisher, USA) coupled to a mass spectrometer (Q-Exactive HFX Orbitrap MS system, Thermo Fisher, CA). In negative mode, BEH C18 column (2.1 × 100 mm,1.7 µm, Waters, USA) is used. Column temperature is 40°C. Gradient starts at 1% mobile phase B with flow rate at 200 μL/min shown as follow: 0–3 min, 1% B; 3–12 min, 1% B to 99% B; 12–17 min, 99% B; 17–17.1 min, 99% B to 1% B; 17.1–20 min, 1% B. Mobile phase A was made by mixing 1L of HPLC-grade water containing 0.3953 g of Ammonium bicarbonate (pH ~8). Mobile phase B contained 100% ACN. The eluted bile acids were inlet to high-resolution MS by negative electrospray ionization mode with a spray voltage of 2,800 V, capillary temperature of 320°C, sheath gas flow rate of 35 arb and auxiliary gas flow rate of 10 arb. The MS analysis was processed with a mass range of *m*/*z* 80–1,200 and a resolution of 60,000 FWHM. Tracefinder 3.2 (Thermo, CA) was used for metabolite identification with in-house library containing MS/MS spectra. Mass tolerance of 10 ppm and 15 ppm was applied for precursor and fragment search. Metabolites were assigned based on fragment matching with MS/MS spectra in the library. Two levels of identification were achieved one with MS/MS confirmation and the other assigned only based on accurate masses of precursor ions. Chromatographic peak area was used for relative quantitation. RT shift of 0.15 min was allowed for peak alignment.

### RNA extraction and RT-qPCR

RNA was extracted from isolated jejunum and liver at steady state or 2 h after oral oil gavage with TRIzol according to the manufacturer’s instructions. Reverse transcription was done with a PrimerScript RT reagent kit (Takara). Quantitative PCR reactions were then carried out with Light Cycler SYBR green DNA master mix (Takara) on an ABI QuantStudio six thermal cycler in triplicate. The following thermal cycling conditions were used: 50°C for 2 min and 95°C for 10 min, followed by 40 cycles of 95°C for 15 s and 60°C for 1 min. The specificity of quantitative PCR was verified with melting curves for each PCR reaction. Primers specific for mouse beta-actin were used as the control to normalize loading. The amount of target mRNA was determined by the difference in cycle threshold (CT) values between the target and loading control. Primers for quantitative PCR are in Table S1.

### EdU pulse-chase 3D migration assays

EdU pulse-chase 3D migration assays have been described in previous reports (Krndija et al., 2019). In brief, mice were i.p. injected with EdU at 10 mg/kg and sacrificed after 24 h, 48 h, or 72 h. Jejunum was isolated from euthanized mice at each specific time point, flushed with PBS, cut into 1 cm long fragments, and fixed in 4% paraformaldehyde for 1 h at RT. The tissues were washed with PBS and then embedded in 4% (*w*/*v*) low-melting point agarose at RT. After agarose polymerization, the jejunums were cut into ~400 μm thick slices by a vibratome (Leica VT1000). Slices were permeabilized with 1% Triton X-100 in PBS for 1 h at RT. EdU detection was performed according to the manufacturer’s instructions for 1 h at RT. Slices were then washed twice with PBS and stained with DAPI (5 μg/mL) for 20 min at RT. Using a 10× lens, confocal z-stacks (Zeiss LSM880) were obtained to capture the whole intestine tissue with *z*-range of at least 50 µm for analysis of cell migration in 3D.

### Immunohistochemical staining of tissue sections

Immunohistochemical staining has been described (Zhang et al., 2015). Tissue slices (6 µm in thickness) from PFA-fixed and paraffin-embedded tissue blocks were mounted on positively charged glass and dewaxed. Antigens were retrieved by boiling the slides in 0.01 mol/L sodium citrate buffer (pH 6.0) for 30 min. The slides were washed with PBS, after which IHC was done with an UltraSensitive TM S-P Kit (Maixin Bio, China). The slides were first treated with hydrogen peroxide for 10 min to block endogenous peroxidase and then incubated with a blocking serum for 10 min at room temperature. The slides were then incubated with rat anti-Ki67 overnight at 4°C, biotinylated secondary goat anti-rat for 10 min at room temperature, and streptavidin-peroxidase for another 10 min at room temperature. Between each incubation step, slides were washed with PBS three times for 5 min. Immunoreactivity was detected with diaminobenzidine. The slides were counterstained with hematoxylin and mounted. Images was captured with a 3DHISTECH SCAN II scanner (3DHISTECH, Budapest, Hungary).

### Immunofluorescence staining of tissue sections

Mice were deeply anaesthetized and transcardially perfused with cold PBS followed by cold 4% PFA and tissues were harvested. Tissues were fixed in 4% PFA overnight, dehydrated and embedded in paraffin. Formalin-fixed and paraffin-embedded tissues were used for immunofluorescence staining as described (Zhang et al., 2015). Briefly, brain or intestine slices (6 μm in thickness) were mounted on positively charged glass and dewaxed. Antigen retrieval was performed by incubation in 0.01 mol/L sodium citrate buffer (pH 6.0) for 30 min in a boiling steamer. Slides were then blocked with 5% BSA for 30 min, followed by sequential incubation with the primary antibodies at 4°C overnight and fluorophore-conjugated secondary antibodies at RT for 2 h. Slides were then counterstained with DAPI (Novon) and mounted in Fluoromount-G (Southern Biotech). Confocal images were obtained with Nikon A1 HD25.

### TUNEL staining

The TUNEL staining was performed using the protocol from the commercial DeadEnd^TM^ Fluorometric TUNEL System kit (Promega, catalog no. G3250). Briefly, deparaffinized and rehydrated sections were fixed in 4% methanol-free formaldehyde solution in PBS for 15 min and then incubated with 100 μL of 20 μg/mL proteinase K for 10 min at RT. Slides were incubated in 4% methanol-free formaldehyde solution in PBS for 5 min and incubated with equilibration buffer for 10 min at RT. The slides were then incubated with 50 μL rTdT enzyme working solution (45 μL equilibration buffer, 5 μL nucleotide mix, and 1 μL rTdT enzyme) at 37°C for 1 h, protected from direct light exposure. Finally, slides were incubated in 2× SSC (saline sodium citrate) for 15 min at RT, washed with PBS, stained with DAPI, and mounted. Images were acquired using Nikon A1 HD25 or Zeiss LSM980 imaging systems.

For samples subjected to both TUNEL staining and immunostaining, immunofluorescence staining was performed first. Slides were incubated with fluorophore-conjugated secondary antibodies at RT for 2 h and washed with PBS for 10 min. The TUNEL staining was subsequently performed using the DeadEnd^TM^ Fluorometric TUNEL System kit protocol.

### Image quantification

Image quantification was performed by a researcher blinded to the identity of images. Numbers of GFAP^+^, Iba1^+^, and TUNEL^+^ cells per field were quantified. Two or four different fields of pons in one individual mouse were captured. A total of 10–18 fields were counted from 4 to 6 individual animals.

The MBP-positive area and MBP immunodensity were quantified using ImageJ software as described (van Tilborg et al., 2017). Briefly, the MBP-positive area was determined by manually setting a threshold to include all MBP-stained tissue, followed by measuring the proportion of the field that was positive for MBP staining. MBP immunodensity was determined by measuring the mean gray value and subtracting the mean value of the background staining. Levels of MBP immunodensity in *Scarb2*^−/−^ mice were normalized to the levels in WT mice, which were set to 100%.

### Extraction of vitamins from plasma and tissues

Mice were fasted for 6 h in the daytime before collecting blood and tissues. Blood was collected via orbital sinus bleeding from anaesthetized mice. Tissues including brain, liver and fat, were collected from euthanized mice.

The plasma was prepared from blood by centrifugation at 3,000 ×*g* for 10 min at 4°C. 100 μL plasma was mixed with 400 μL ice-cold extraction solution (methanol: isopropanol = 4:1, *v*/*v*) to precipitate proteins and extract vitamins. The mixture was vortexed for 30 s and sonicated for 15 min, followed by centrifugation at 16,000 ×*g* for 20 min at 4°C. The supernatant was evaporated and resuspended in 50 μL methanol for UPLC-MS analysis. Light was avoided throughout the preparation.

50 mg tissue was added into 1 mL 80% methanol (methanol: ddH_2_O = 4:1, *v*/*v*, containing 0.5 mg ascorbic acid) and homogenized on ice. The mixture was vortexed for 30 s and sonicated for 30 min at 4°C, followed by centrifugation at 16,000 ×*g* for 20 min at 4°C. 500 μL supernatant was transferred to a new tube. The supernatant was evaporated and resuspended in 50 μL methanol for UPLC-MS analysis.

### UPLC-MS analysis of vitamin

Methanol extract of 5 μL from vitamin extraction preparations and reference standards were analyzed using ultra-high pressure liquid chromatography (ACQUITY^TM^ UPLC system, Waters, USA) coupled to a quadrupole orbitrap mass spectrometer (Q-Exactive Plus^TM^ Orbitrap MS system, Thermo Scientific, USA). LC separation was performed on a reverse-phase BEH C_18_ column (50 mm × 2.1 mm, 1.7 µm; Waters ACQUITY^TM^, USA) equipped with a VanGuard pre-column (5 mm × 2.1 mm, 1.7 µm; Waters, USA). The LC elution gradient using a mobile phase of solvent A (0.1% formic acid in ultra-pure water) and solvent B (methanol), which was optimized as follows: 0–4 min, 2% to 100% B; 4–8 min, 100% B; 8–9 min,100% to 2% B; and 9–10 min, 2% B. The flow rate was 0.2 mL/min and the column temperature was maintained at 40°C. The eluted vitamin extracts were inlet to high-resolution MS (HRMS) by positive electrospray ionization (ESI^+^) with an ion-spray voltage of 3,800 V, vaporiser temperature of 350°C, capillary temperature of 320°C, auxiliary gas heater temperature of 300°C, sheath gas flow rate of 35 arb and auxiliary gas flow rate of 10 arb. The MS analysis was conducted with a resolution of 70,000 full width at half maximum (FWHM), automatic gain control (AGC) target of 5e^5^, maximum inject time of 100 ms and stepped normalized collision energy (NCE) of 35 V. The isolation window was controlled within *m*/*z* 2.0. Targeted-selected ion monitoring/data dependent MS/MS (T-SIM/dd-MS^2^) and parallel reaction monitoring (PRM) scanning mode were employed for the qualitative and quantitative analysis of vitamin E (*m*/*z* 431.3884), vitamin A1 (*m*/*z* 287.2369), 25-hydroxyl vitamin D2 (*m*/*z* 413.3414), 25-hydroxyl vitamin D3 (*m*/*z* 401.3414), vitamin K1 (*m*/*z* 451.3571), vitamin B3 (*m*/*z* 123.0553), vitamin C (*m*/*z* 177.0394) and vitamin B12 (*m*/*z* 1,355.5747). Xcalibur 4.1.5 and Tracefinder (Thermo Scientific, USA) software were utilized to acquire and process MS and MS/MS data for corresponding peak abundance. All the samples were randomly injected during data acquisition.

### Statistical analysis

The data are presented as the mean ± SEM unless otherwise stated. All statistical analyses were conducted with GraphPad Prism version 8.0. For comparisons of two groups, an unpaired two-tailed Student’s *t*-test was used. For comparisons of more than two groups, one-way or two-way ANOVA was used to judge the impact of each independent category on the experimental outcome. All *P* values less than 0.05 were considered significant and are indicated in the figures with 1–4 asterisks: *P < 0.05, **P < 0.01, ***P < 0.001, ****P < 0.0001. Nonsignificant differences are indicated by ns.

## Supplementary data

The online version contains supplementary material available at https://doi.org/10.1093/procel/pwae016.

pwae016_suppl_Supplementary_Figures_S1-S8_Tables_S1

## Data Availability

16S rRNA gene sequencing data have been submitted to NCBI Sequence Read Archive (SRA) with the accession number of PRJNA979172. Other data or materials generated in this study are available from the corresponding authors upon request.

## Code availability

All the custom codes are available from the corresponding authors upon reasonable request.
