# Supplementary material for: Gut dysbiosis impairs intestinal renewal and lipid absorption in Scarb2 deficiency-associated neurodegeneration
Source: Protein Cell. 2024 Apr 18;15(11):818–39. doi: 10.1093/procel/pwae016 (PMC11528516; doi:10.1093/procel/pwae016)
Supplement: pwae016_suppl_Supplementary_Figures_S1-S8_Tables_S1 [file pwae016_suppl_supplementary_figures_s1-s8_tables_s1.pdf]

Supplemental Materials

Supplemental Figure and Figure Legends

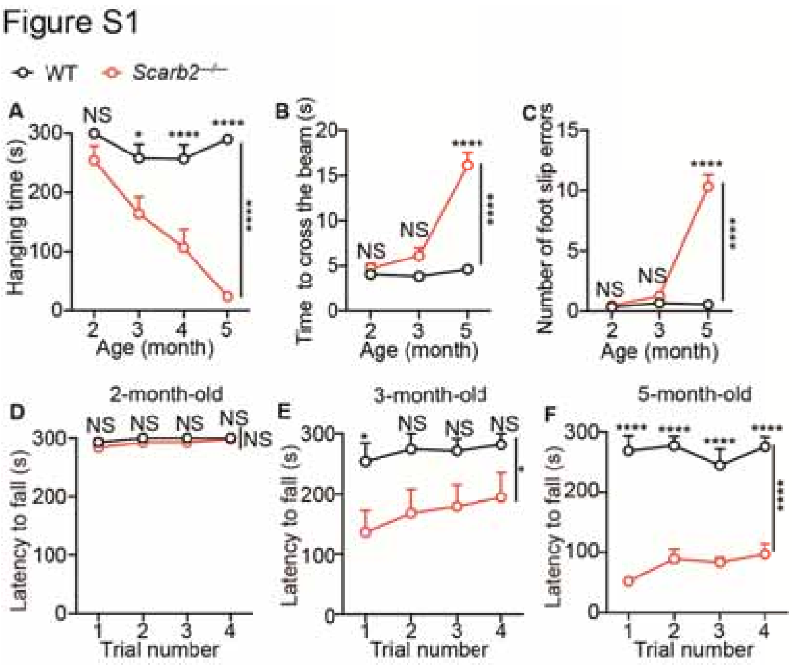

Figure S1. ***Scarb2*$^{-/-}$ mice develop age-dependent movement impairments.** (**A**) Hanging time of the indicated mice at different ages (n=8 mice per group). (**B**, **C**) Time to cross the beam (**B**) and number of foot slip errors (**C**) in the beam cross test in WT and *Scarb2*$^{-/-}$ mice at different ages (n=8 mice per group). (**D**-**F**) Latency to fall, measured by rotarod test, of the indicated mice at different ages (n=8 per mice group). Means with bars indicating s.e.m. are plotted in (**A**-**F**). *P* values were calculated using two-way ANOVA with the Bonferroni correction for multiple tests in (**A**-**F**). NS indicates no significant difference (*P*>0.05). **P*<0.05, *******P*<0.0001.

Table S1 Primers related to Methods

Gene Name	Sequences 5’-3’
FXR	TCCAGGGTTTCAGACACTGG
	GCCGAACGAAGAAACATGG
FGF15	ACGTCCTTGATGGCAATCG
	GAGGACCAAAACGAACGAAATT
Shp	CGATCCTCTTCAACCCAGATG
	AGGGCTCCAAGACTTCACACA
Ibabp	CAGGAGACGTGATTGAAAGGG
	GCCCCCAGAGTAAGACTGGG
Ostalpha	TTGTGATCAACCGCATTTGT
	CTCCTCAAGCCTCCAGTGTC
Asbt	TTGCACAGCACAAGCAGTGA
	TGCATTGAAGTTGCTCTCAGGT
CD36	GCCAAGCTATTGCGACATGA
	ATCTCAATGTCCGAGACTTTTCAAC
FABP2	TGCGAACTGGAGACCATGAC
	TCAGTCACGGACTTTATGCCT
FATP4	CCAGTAGTGTGGCCAACTTCCT
	CCACAGACCCACAAACTCATTG
DGAT1	TTTCAGCAATTATCGTGGTATCC
	AAAAATAACCTTGCATTACTCAGGA
DGAT2	GCGCTACTTCCGAGACTACTT
	GGGCCTTATGCCAGGAAACT
ACAT2	AGACTTGGTGCAATGGACTCGAC
	CATAGGGCCCGATCCAACAG
APOB	TGAATGCACGGGCAATGA
	GGCATTACTTGTTCCATGGTTCT
APOA4	CACACAGACCCAGGAAATGA
	CCTTGATCGTGGTCTGCAT
MTTP	ATGATCCTCTTGGCAGTGCTT
	TGAGAGGCCAGTTGTGTGAC
Plin2	CACTCCACTGTCCACCTGATT
	TCCTGAGCACCCTGAATTTT
Cyp7a1	AGCAACTAAACAACCTGCCAGTACTA
	GTCCGGATATTCAAGGATGCA
Cyp8b1	GGCTGGCTTCCTGAGCTTATT
	ACTTCCTGAACAGCTCATCGG
Cyp7b1	GAACCGATCGAACCTAAATTCCT
	TAGCCCTCTTTCCTCCACTCATA
MafG	GACCCCCAATAAAGGAAACAA
	TCAACTCTCGCACCGACA
Gapdh	AGGTCGGTGTGAACGGATTTG
	TGTAGACCATGTAGTTGAGGTCA
